# A proteomic analysis of the functional effects of fatty acids in NIH 3T3 fibroblasts

**DOI:** 10.1186/1476-511X-10-218

**Published:** 2011-11-24

**Authors:** Juliana Magdalon, Elaine Hatanaka, Talita Romanatto, Hosana G Rodrigues, Wilson MT Kuwabara, Caitriona Scaife, Philip Newsholme, Rui Curi

**Affiliations:** 1 Institute of Biomedical Sciences, University of Sao Paulo, Sao Paulo, Brazil; 2 Institute of Physical Activity and Sport Sciences, University Cruzeiro do Sul, Sao Paulo, Brazil; 3 School of Biomolecular and Biomedical Science, University College Dublin, Dublin, Ireland; 4 School of Biomedical Sciences, Curtin University, Perth, Western Australia 6845, Australia

**Keywords:** oleic acid, linoleic acid, palmitic acid, enolase, FBP, c-myc, protein expression, proliferation

## Abstract

Previous studies have demonstrated that long chain fatty acids influence fibroblast function at sub-lethal concentrations. This study is the first to assess the effects of oleic, linoleic or palmitic acids on protein expression of fibroblasts, as determined by standard proteomic techniques. The fatty acids were not cytotoxic at the concentration used in this work as assessed by membrane integrity, DNA fragmentation and the MTT assay but significantly increased cell proliferation. Subsequently, a proteomic analysis was performed using two dimensional difference gel electrophoresis (2D-DIGE) and MS based identification. Cells treated with 50 μM oleic, linoleic or palmitic acid for 24 h were associated with 24, 22, 16 spots differentially expressed, respectively. Among the identified proteins, α-enolase and far upstream element binding protein 1 (FBP-1) are of importance due to their function in fibroblast-associated diseases. However, modulation of α-enolase and FBP-1 expression by fatty acids was not validated by the Western blot technique.

## Introduction

Found in the majority of tissues of the body, fibroblasts are responsible for the synthesis and secretion of most extracellular matrix (ECM) components, such as proteoglycans, collagens, laminin and fibronectin, which bind to proteins expressed on cell surfaces thus modulating physiologic responses. Fibroblasts can also secrete proteinases, including matrix metalloproteinases and plasminogen, hence playing an important role in ECM degradation and tissue remodeling [1-3].

The dysregulation of fibroblast biology is associated with several diseases and pathological states, including deficient wound healing [4-6], pulmonary diseases [7,8], cardiovascular diseases [9,10] and cancer [11]. Therefore, the discovery of new therapies modulating fibroblast biology could be a powerful target to develop treatment against such diseases. Oleic (18:1n-9), linoleic (18:2n-6) and palmitic (16:0) acids are the most abundant fatty acids in the western diets [12]. The latter is a saturated fatty acid found in palm oil, butter, milk, cheese and meats, whereas oleic acid is a monounsaturated fatty acid found in olive oil, meat, eggs and milk and linoleic acid is a polyunsaturated fatty acid found in soybean, sunflower, safflower and corn oils. The effects of prostaglandin derived from n-3 and n-6 fatty acids on specific protein expression (e.g. COX-2 and IL-6) in NIH 3T3 fibroblasts [13] and some aspects of the impact of fatty acids on process involving fibroblast function [14-16] have been reported. However, no systematic study has assessed the pleiotropic effects of fatty acids on fibroblast protein expression. Therefore, the aim of this study was to assess the effects of oleic (OLA), linoleic (LNA) or palmitic acids (PAM) on protein expression of NIH 3T3 fibroblasts, as determined by 2D-DIGE, i.e., separation of proteins by isoelectric focusing in the first dimension followed by SDS-PAGE in the second. This approach has been successful in elucidating the actions of palmitic acid on protein expression in a clonal pancreatic beta cell line, INS-1E [17].

## Results

### Effect of fatty acids on fibroblast membrane and DNA integrity

**Figure 1:**
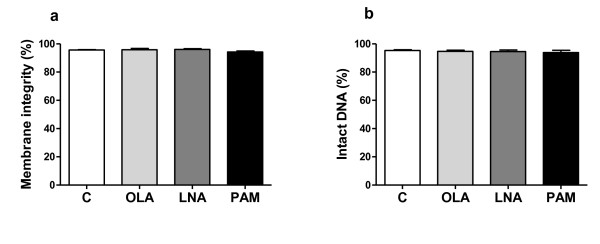
**Analysis of membrane integrity (a) and DNA fragmentation (b): NIH 3T3 fibroblasts (10^5 ^cells) were treated with vehicle control (C), 50 μM OLA, LNA or PAM for 24 h**. Results are presented as mean ± standard error of the mean (SEM) of at least 4 (membrane integrity) or 8 (intact DNA) independent samples.

Fibroblast cells were incubated in the presence 50 μM oleic, linoleic or palmitic acids for 24 h. Membrane integrity and DNA fragmentation assays indicated that the fatty acids were not toxic to the cells at this concentration (Figure 1).

### Effect of fatty acids on relative cell number as assessed by MTT assay

**Figure 2:**
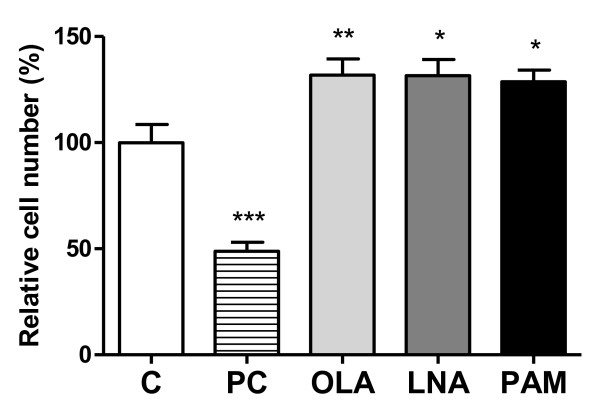
**Analysis of relative cell number using the MTT assay: NIH 3T3 fibroblasts (6 × 10^3 ^cells) were treated with vehicle control (C), 300 μM palmitic acid (positive control - PC), 50 μM OLA, LNA or PAM for 24 h**. Results are presented as mean ± (SEM) of at least 7 independent samples. *p < 0.05, **p < 0.01 and ***p < 0.001 comparing to control (ANOVA and Dunnett post hoc test).

The treatment of fibroblasts with 50 μM oleic, linoleic or palmitic acids increased relative cell number by 1.32-fold (*p *< 0.01), 1.32-fold (*p *< 0.05) or 1.29-fold (*p *< 0.05), respectively, as assessed by MTT absorbance (Figure 2). However, the treatment with 300 μM palmitic acid (known to be cytotoxic) decreased relative cell number by 50% (Figure 2).

### 2D-DIGE and MS based protein identification

**Figure 3:**
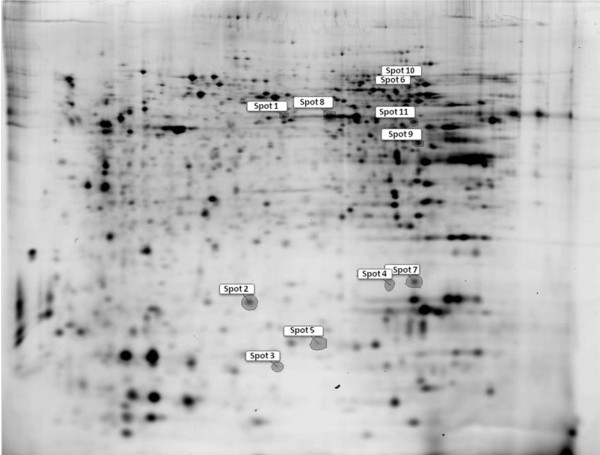
**Analysis of protein expression using 2D-DIGE: The figure is a representative image of the gel with the excised and identified spots**.

Using 2D-DIGE and Progenesis Samespots v 3.2 analyses software, a protein profile from control or fatty acid (oleic, linoleic or palmitic acids) treated fibroblasts was assessed. Comparing protein expression using Student's *t *test, cells treated with oleic, linoleic and palmitic acid showed 24, 22, 16 spots significantly differentially expressed (*p *< 0.05), respectively, when compared to control, whereas 9, 8, 6 of them were associated with a power > 0.8, respectively. According to spot intensity and matching from DIGE to preparative gels, 11 spots were chosen to be identified using MS (Figure 3). The majority of the spots identified contained one or two proteins when data was searched against the Swiss Prot mouse databases using Turbosequest. However, the identified proteins were not matched against their position in the gel according to their molecular weight and isoeletric point (Table 1).

**Table 1:** Proteins are described followed by accession number and the number of peptides identified using mass spectrometry of excised spots.

Spot	Identified proteins, accession number and number of peptides	Fatty acid (FA)	Control/FA ratio	Statistics
**1**	α-enolase (P17182 - 11 peptides); Mitochondrial processing peptidase subunit beta (Q9CXT8 - 3 peptides)	OLA LNA	1.1 1.1	*p *< 0.05; power > 0.8 *p *< 0.05; power > 0.8

**2**	26S protease regulatory subunit 8 (P62196 - 12 peptides)	LNA OLA PAM	1.1 1.1 1.1	*p *< 0.05; power > 0.8 *p *< 0.05 *p *< 0.05

**3**	Histone H$ (P62806 - 2 peptides); Histone H1.2 (P15864 - 2 peptides)	LNA	0.9	*p *< 0.05; power > 0.8

**4**	ATP synthase subunit beta (P56480 - 4 peptides); Eukaryotic translational initiation factor 5A (P63242 - 2 peptides)	OLA	1.2	*p *< 0.05; power > 0.8

**5**	Nothing identified	OLA PAM	1.1 1.1	*p *< 0.05; power > 0.8 *p *< 0.05

**6**	Heterogeneous nuclear ribonucleoprotein L (Q8R081 - 9 peptides); Lamin A/C (P48678 - 7 peptides)	OLA	1.1	*p *< 0.05; power > 0.8

**7**	FK506-binding protein 4 (P30416 - 6 peptides); Transcription factor BTF3 (Q64152 - 3 peptides)	PAM OLA	1.1 1.1	*p *< 0.05; power > 0.8 *p *< 0.05

**8**	Heterogeneous nuclear ribonucleoprotein (Q8R081 - 2 peptides)	PAM OLA	1.1 1.1	*p *< 0.05; power > 0.8 *p *< 0.05

**9**	α-enolase (P17182 - 10 peptides)	PAM	1.1	*p *< 0.05; power > 0.8

**10**	α-enolase (P17182 - 10 peptides); 40 kDa Peptidyl prolyl cis-trans isomerase (Q9CR16 - 4 peptides)	PAM OLA	1.1 1.05	*p *< 0.05; power > 0.8 *p *< 0.05

**11**	α-enolase (P17182 - 5 peptides); Far upstream element-binding protein 1 (Q91WJ8 - 4 peptides)	PAM	1.1	*p *< 0.05; power > 0.8

Where appropriate, fatty-acid treatment that significantly changed protein expression is indicated (*p *< 0.05). Abbreviations: LNA (linoleic acid), OLA (oleic acid) and PAM (palmitic acid).

### Protein analysis by Western blot

**Figure 4:**
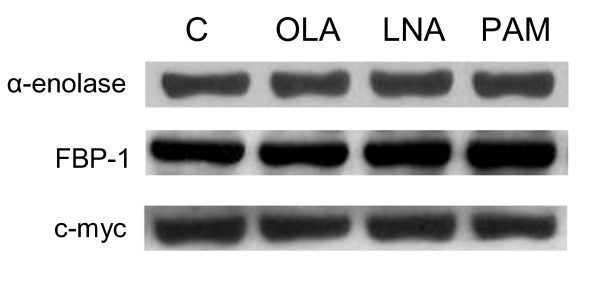
**Analysis of protein expression using the Western Blot technique: Effects of oleic, linoleic or palmitic acid on α-enolase, FBP-1 and c-myc expression**. The analysis was performed by Western blot using 40 μg protein from NIH 3T3 fibroblasts treated with vehicle control (C), 50 μM OLA, LNA or PAM for 24 h. Representative Western blot of six independent experiments. β-Actin was used as an expression control.

Based on results obtained using 2D-DIGE and MS we selected α-enolase and FBP-1 due to their functional importance in fibroblast-associated diseases. Therefore, western blotting was performed in order to validate our previously described results (Table 1). In addition, c-myc expression was assessed since FBP-1 is a known modulator (see discussion). The expression of these three proteins was not altered by treatment with fatty acids (Figure 4).

## Discussion

In order to fully comprehend a biological network, the expression information from mRNA and concentration change in protein is required. The importance of proteomic analysis is emphasised when differences between the relative expression levels of mRNA and their corresponding proteins are demonstrated [18]. Several studies have analysed the effects of fatty acids on the gene expression profile of different cell and tissue types [19-24] but relatively few have studied such effects based on proteomic approaches. Our study is the first to investigate the effects of oleic (monounsaturated), linoleic (polyunsaturated) or palmitic (saturated) acids on protein expression of murine fibroblasts and related this to a functional outcome, cell proliferation.

To test the cytotoxicity or proliferative activity of the fatty acids, NIH 3T3 cells were treated with 50 μM fatty acids over 24 hours and both cell viability and relative cell number were assessed using flow cytometry and MTT assay, respectively. This concentration is able to cause biological effects on other cell types [25-27] and is in the physiological range for plasma free fatty acids [28,29]. There was no difference between control and fatty acid treated cells based on membrane integrity and DNA fragmentation measurements, although results from the MTT assay indicated an increase in relative cell number (proliferation) in cells treated with 50 μM oleic, linoleic or palmitic acids. Oleic and linoleic acids increase skeletal muscle cell proliferation, whereas palmitic acid does not affect it [30]. Moreover, oleic and linoleic acids promote proliferation of epithelial cells [31].

Subsequently, a proteomic analysis was performed using 2D-DIGE. Cells treated with oleic, linoleic or palmitic acid for 24 h were associated with 24, 22, 16 spots differentially expressed, respectively, and 11 of them were identified by mass spectrometry using LC/MS/MS. Among the identified proteins, α-enolase and FBP-1 are of importance due to their function in fibroblast-associated diseases. In addition to its role in glycolysis, α-enolase is a cell-surface plasminogen-binding site forming plasmin, which is responsible for degradating extracellular matrix compounds, such as collagen, fibrin, fibronectin and laminin [32]. Therefore, signaling initiated by plasminogen binding to the cell surface has important implications for physiological and pathological events, such as wound healing, tissue remodeling and tumor cell diffusion [33]. FBP modulates c-myc expression through binding to FUSE, that is upstream to P2 promoter [34-37]. The transcription factor c-myc plays a role in the regulation of approximately 10% of cell genes and is involved in proliferation and metabolism and hence it is considered a proto-oncogene [38,39].

We observed minor discrepancies between 2D gel and Western blot results which describe the fatty acid dependent changes in expression level of two specific proteins. However, differences in protein expression using the latter techniques have also been reported by other groups [17,40]. The discrepancy may be explained by the fact that the Western blot technique cannot separate different forms of proteins with specific post-translational modifications (for example, glycosylation), whereas 2D gels are able to detect differences, therefore 2D gel analysis is more precise. However, it should be noted that the analysis from mass spectrometry in the present study identified two proteins in some spots, thus the use of Western blot was important for confirmation purposes.

In conclusion, although previous studies have reported potent effects of fatty acids on fibroblast, our results suggest that changes in protein expression using 50 μM oleic, linoleic or palmitic acids for 24 h were relatively minor. However, it should be noted that relatively small changes in protein expression can lead to a significant change in cell proliferation.

## Materials and methods

### Chemicals and reagents

Oleic, linoleic and palmitic acids, Cell Growth Determination Kit MTT Based and Propidium Iodide were purchased from Sigma-Aldrich (St. Louis, MO, USA). Dulbecco's Modified Eagle Medium (DMEM), Penicillin Streptomycin (Pen Strep) and Fetal Bovine Serum (FBS) were purchased from Invitrogen (Carlsbad, CA, USA). Antibodies against α-enolase and c-myc were purchased from Cell Signaling Technology (Beverly, MA, USA) and antibody against FBP-1 was from Santa Cruz Biotechnology (Santa Cruz, CA, USA).

### Culture conditions

NIH 3T3 fibroblasts were cultured in DMEM medium supplemented with 10% FBS, 100 U/mL Penicilin and 100 μg/mL Streptomycin and were maintained in a humidified incubator at 37°C in an atmosphere of 5% CO_2_. After 3 h of incubation (for all the experiments except Western blot), the samples were washed with PBS (KCl 2.7 mM, NaCl 136.8 mM, Na_2_HPO_4 _anhydrous 6.4 mM, KH_2_PO_4 _0.9 mM - pH 7.4) and treated with 50 μM oleic, linoleic or palmitic acids in the above medium for 24 h. Ethanol was used as control treatment and its final concentration did not exceed 0.16%.

### Fatty-acid cytotoxicity as determined by membrane integrity and DNA fragmentation

In order to evaluate fatty-acid cytotoxicity, membrane integrity and DNA fragmentation were measured using flow cytometry. Fibroblasts (10^5 ^cells) were incubated in 6-well plates in a final volume of 1 mL. After 24 h of treatment with the fatty acids, the cells were harvested using trypsin, followed by centrifugation at 500 g. For membrane integrity assay, cells were then re-suspended in 200 μL PBS and 20 μL propidium iodide solution (20 μg/mL in PBS) was added to each sample. For DNA fragmentation, cells were resuspended in 200 μL lysis buffer (0.1% sodium citrate and 0.1% Triton X-100) with 20 μg/mL propidium iodide and were then incubated in dark for 30 minutes. Subsequently, cells were analysed (8000 cells/sample) in a flow cytometer (FACSCalibur, Becton Dickinson, San Juan, CA, USA) using Cell Quest software. Propidium iodide is a water-soluble fluorescent compound that cannot pass through intact plasma membrane. Therefore, when plasma membrane is disrupted, propidium iodide enters the cells and intercalates between DNA bases, which is identified by high fluorescence emission. Likewise, in the DNA fragmentation assay, propidium iodide intercalates in the exposed DNA after cell lysis and the presence of low-fluorescent fragments is indicative of DNA fragmentation. Fluorescence was measured using FL2 channel (orange-red fluorescence-585/42nm).

### Relative cell number as determined by the MTT assay

MTT assay (Sigma Aldrich, St. Louis, MO, USA) is a colorimetric assay that indirectly measures cellular dehydrogenase enzyme activity, which is mainly associated with functional mitochondria. The MTT salt in the oxidized form is yellow and water-soluble and can be reduced, for example, by mitochondrial succinate dehydrogenase present in viable cells to formazan crystals. An increase in the number of viable cells results in an increase in the overall activity of mitochondrial dehydrogenases in the sample, for example, as assessed by the WST-1 assay [41]. Fibroblasts (6 × 10^3 ^cells) were incubated in 96-well plates in a final volume of 100 μL. For positive control (higher death rate), 300 μM palmitic acid was used. After 24 h of treatment with the fatty acids, the supernatants were discarded and 100 μL 0.1 N HCl in anhydrous isopropanol was added in order to dissolve the formazan crystals. After dissolution, sample absorbance was measured in spectrophotometer (570 nm and 690 nm) and plotted as the difference between the results found in both wavelengths, as specified by the supplier.

### Sample preparation for 2D-DIGE

Cells were incubated in 100% confluence in a final volume of 1 mL. After 24 h of treatment with the fatty acids, samples were harvested. For harvesting the cells, the supernatant was removed and the cells were carefully washed four times in an isotonic sucrose solution (0.35 M). A lysis buffer (9.5 M urea; 2% CHAPS) was added and three wells pooled for each sample. Subsequently, the samples were sonicated and centrifuged at 500 g in a microcentrifuge. The supernatant was collected and the protein concentrations were determined using a modified protein assay [42]. The samples were then stored at -80°C.

### CyDye labeling

In order to run 2D-DIGE, the samples were previous labeled with fluorescent dyes. A CyDye (Amersham, GE Healthcare) stock solution (1 mM) was made adding anhydrous DMF and stored at -20°C. The Cy3 (Amersham CyDye DIGE Fluor Cy3 minimal dye, GE Healthcare, 25-8008-61) and Cy5 (Amersham CyDye DIGE Fluor Cy5 minimal dye, GE Healthcare, 25-8008-62) solutions were prepared (400 μM) using the CyDye stock solution and anhydrous dimethylformamide (DMF).

After thawing, each sample was adjusted to a final concentration of 5 μg/μL and pH between 8 and 9. Forty microgram of protein from fifteen samples (three from each group sample: control, oleic, linoleic and palmitic) were independently labeled with 0.8 μL of Cy5 solution. A pool standard was then prepared by mixing fifty-five microgram of all fifteen samples and the resulting mixture was labeled with 16.5 μL of Cy3. All the samples, including the pool standard, were kept in the dark for 30 min and the reaction was then stopped by the addition of lysine (10 mM) for 10 min on ice. DIGE dilution buffer (9.5 M Urea; 2% CHAPS; 2% DTT; 1.6% Pharmalyte) was added to each sample so that the final volume was doubled.

Fifteen mixtures were prepared, each made of one sample labeled with Cy5 and the pool standard labeled with Cy3. These mixtures were stored at -80°C for subsequent running independent 2D gels.

### 2D-DIGE

Rehydration buffer (8 M Urea; 0.5% CHAPS; 0.2% Pharmalyte; 0.2% DTT) was added to each sample so that the final volume was 450 μL. Strips (24 cm, pH 3-10, non-linear) were then rehydrated overnight with such solutions and covered with IPG Cover Fluid (Amersham, 17-1335-01) to avoid dehydration. After rehydration, the strips were placed in the Ettan IPGphor Manifold, covered with dry strip cover fluid. The isoeletric focusing was carried out on the IPGphor III IEF system (GE Healthcare) according to the following parameters: Step, 3500 V, 7500 vHrs; Gradient, 8000 V, 10 minutes; Step, 8000 V, 1 h; Step, 100 V, 12 h. Subsequently, the strips were equilibrated for 15 minutes in equilibration buffer (6 M Urea, 50 mM TrisCl pH 8.8, 30% (v/v) Glycerol, 2% (w/v) SDS) containing 1% (w/v) DTT and 15 min in equilibration buffer containing 2.5% (w/v) iodoacetamine. The strips were then briefly washed in SDS electrophoresis buffer (25 mM Tris; 192 mM Glycine; 0.1% (w/v) SDS) and placed on the top of 1 mm gels previously prepared (National Diagnostics ProtoGel kit, 30% acrylamide). 1% w/v agarose solution was poured on the top of each gel in order to seal and fix the strip on the gel. Following, second dimension separation was performed in a Protean Plus Dodeca cell (Bio-Rad, UK) with SDS electrophoresis buffer at 15°C. The gels were run at 0.2 W/gel for 1 h, followed by 1 W/gel overnight. Gel images were scanned with a Typhoon 9410 Variable Mode Imager (GE Healthcare) and analysed using Progenesis Samespots v 3.2. A significant difference between control and fatty-acid treated samples was set at *p *< 0.05 and power > 0.8 when analysed by Student's *t*-test.

### Sample preparation for mass spectrometry analysis

Eleven protein spots, which were significantly differentially expressed between control and treated cells, were excised from preparative gels, which were silver-stained using a commercial kit (Silver Stain PlusOne; Amersham Pharmacia Biotech, Amersham, UK). The silver-stained spots of interest were manually excised, placed into 1.5 mL tubes and washed in ddH_2_0 at 4°C overnight. The gel plugs were then destained and dehydrated, followed by their protein digestion and peptide extraction. For destaining, an equal volume of 30 mM potassium ferricyanide and 100 mM sodium thiosulphate was added to the gel plugs and incubated at room temperature with occasional shaking until the brown colour had disappeared. The supernatants were discarded and the plugs were washed three times with ddH_2_0. They were then incubated in 200 mM NH_4_HCO_3 _for 20 min at room temperature on a thermomixer. For dehydrating, the supernatants were removed and replaced with 200 mM NH_4_HCO_3_:ACN::2:3 solution. The gel plugs were incubated at 37°C on a thermomixer for 10 min with gentle shaking. After briefly spinning, the supernatants were discarded and the plugs were incubated with 70 μl of 50 mM NH_4_HCO_3 _at 37°C on a thermomixer for 10 min with gentle shaking. After briefly spinning, the supernatants were discarded and gel plugs were dried at 37°C on a Speedivac until they were white in colour. For digesting the proteins, the gel plugs were rehydrated in trypsin solution (Promega, UK, 20 ng/μl in 25 mM NH_4_HCO_3_, pH 8.4) on ice for 45 min. The supernatants were removed and the gel plugs covered with 25 mM NH_4_HCO_3_, pH 8.4, and incubated at 37°C overnight on a thermomixer. For extracting the peptides, the supernatants were saved in separate tubes and the tryptic peptides were extracted three times with 50 μl of 70% acetonitrile, 5% formic acid. Each extraction was sonicated for 5 min and the supernatants from the same sample were combined. These were then dried in a Speedivac and stored at -20°C for subsequent protein identification using mass spectrometry.

### Mass spectrometry (MS) analysis

The proteins were identified by Thermo Finnegan LTQ mass spectrometer, using LC/MS/MS. All of the data was searched against the Swiss Prot mouse databases using Turbosequest. Peptides with a probabililty of >0.001 were discounted.

### Western blot

Fibroblasts (10^6 ^cells) were incubated in 75 mm^2 ^flasks in a final volume of 10 mL. After 24 h, supernatants were discarded and the cells were incubated with 50 μM of oleic, linoleic or palmitic acids in DMEM (10% FBS) in a final volume of 12 mL for 24 h. Subsequently, supernatants were discarded and flasks were washed two times with ice-cold PBS. For protein extraction, 250 μL extraction buffer (10 mM EDTA, 100 mM TRIS, 10 mM sodium pyrophosphate, 100 mM sodium fluoride, 10 mM sodium vanadate, 2 mM PSMF, 3% aprotinin, 1% Triton X-100) was added, the cells were scraped and then harvested. After 10 min of shaking in 4°C, samples were centrifuged (11000 rpm, 15 min, 4°C) and the proteins in the supernatants were quantified by the Bradford method. Subsequently, Laemmli sample buffer with 200 mM dithiothreitol (DTT) was added to the samples. They were then boiled for 5 min and frozen at -80°C.

For immunoblotting, the samples were boiled for 5 min and 40 μg of total protein was separated by SDS-PAGE, transferred to nitrocellulose membranes, and blotted with anti-enolase-1 (Cell Signaling Technology, #3810), anti-FBP-1 (Santa Cruz Biotechnology Inc, SC-11101), anti-c-Myc (Cell Signaling Technology, #9402) and anti-β-Actin (Cell Signaling Technology, #4967). Subsequently, the blots were incubated with HRP-conjugate antibodies for 2 h and bands were detected by chemiluminescence using Amersham Biosciences (RPN 2209), with following exposure of the membranes to radiographic films. β-Actin was used as an expression control.

### Statistical analysis

For all the experiments except 2D-DIGE, the comparison between control and samples treated with oleic, linoleic or palmitic acids were performed using ANOVA and Dunnett post hoc test. For 2D-DIGE, the comparison between control and samples treated with oleic, linoleic or palmitic acids were performed using Student's *t *test.

## Abbreviations

2D-DIGE: two dimensional difference gel electrophoresis; ECM: extracellular matrix; FBP-1: far upstream element binding protein 1; FUSE: far upstream element; LNA: linoleic acid; OLA: oleic acid; PAM: palmitic acid.

## Competing interests

The authors declare that they have no competing interests.

## Authors' contributions

JM conceived the study, participated in its design, acquisition of data, statistical analysis and drafted the manuscript; EH conceived the study and participated in its design; TR participated in the Western Blotting; HGR participated in the MTT assay; WMTK participated in the Western Blotting; CS participated in the 2D-DIGE and carried out mass spectrometry analysis; PN participated in the design of the study and helped to draft the manuscript; RC conceived the study, participated in its design and coordination and helped to draft the manuscript. All authors read and approved the final manuscript.
